# ER/K linked GPCR-G protein fusions systematically modulate second messenger response in cells

**DOI:** 10.1038/s41598-017-08029-3

**Published:** 2017-08-10

**Authors:** Rabia U. Malik, Matthew Dysthe, Michael Ritt, Roger K. Sunahara, Sivaraj Sivaramakrishnan

**Affiliations:** 1 Department of Genetics, Cell Biology, and Development, University of Minnesota, Minneapolis, MN 55455 USA; 2 Department of Pharmacology, University of California, San Diego, La Jolla, CA 92093 USA

## Abstract

FRET and BRET approaches are well established for detecting ligand induced GPCR-G protein interactions in cells. Currently, FRET/BRET assays rely on co-expression of GPCR and G protein, and hence depend on the stoichiometry and expression levels of the donor and acceptor probes. On the other hand, GPCR-G protein fusions have been used extensively to understand the selectivity of GPCR signaling pathways. However, the signaling properties of fusion proteins are not consistent across GPCRs. In this study, we describe and characterize novel sensors based on the Systematic Protein Affinity Strength Modulation (SPASM) technique. Sensors consist of a GPCR and G protein tethered by an ER/K linker flanked by FRET probes. SPASM sensors are tested for the β2-, α1-, and α2- adrenergic receptors, and adenosine type 1 receptor (A_1_R), tethered to Gαs-XL, Gαi_2_, or Gαq subunits. Agonist stimulation of β2-AR and α2-AR increases FRET signal comparable to co-expressed FRET/BRET sensors. SPASM sensors also retain signaling through the endogenous G protein milieu. Importantly, ER/K linker length systematically tunes the GPCR-G protein interaction, with consequent modulation of second messenger signaling for cognate interactions. SPASM GPCR sensors serve the dual purpose of detecting agonist-induced changes in GPCR-G protein interactions, and linking these changes to downstream signaling.

## Introduction

G protein coupled receptors relay detection of stimuli such as photons, neurotransmitters, hormones, or drugs from the extracellular milieu to the intracellular environment by binding and activating one or more functionally distinct heterotrimeric G proteins^1^. Coupling between an active GPCR and G proteins stimulates accumulation of second messengers such as cyclic AMP, calcium/potassium ions, and inositol phosphate^1^. Efforts to monitor and understand the link between GPCR-G protein interactions and downstream second messenger response are complicated by the range of cellular factors that influence GPCR signaling including the relative localization and abundance (concentration) of GPCR and G proteins, regulatory proteins such as Regulators of G protein Signaling (RGS), G protein kinases (GRKs), and non-G protein effectors such as β-arrestins^2^.

Current approaches to visualize GPCR-G protein interaction in cells involve probing the interaction between individually expressed fluorescent or luminescent protein fusions using fluorescence/bioluminescence resonance energy transfer (FRET/BRET)^3–7^. Therefore, insights into the GPCR-G protein interaction gained from these studies are limited by their dependence on the relative concentration and co-localization of individually expressed GPCR and G protein. Alternatively, direct GPCR-G protein fusions generated by tethering the N-terminus of the Gα to the GPCR’s C-tail either directly or with a short linker in between have been used to study the influence of tethering different G protein subunits on G protein activation and second messenger signaling^8, 9^. Such fusions have not been used to monitor the GPCR-G protein interaction using resonance energy transfer approaches^6^. A limitation of direct fusions is that GPCRs with short C-tails do not efficiently signal to the tethered G protein^10–13^.

**Figure 1:**
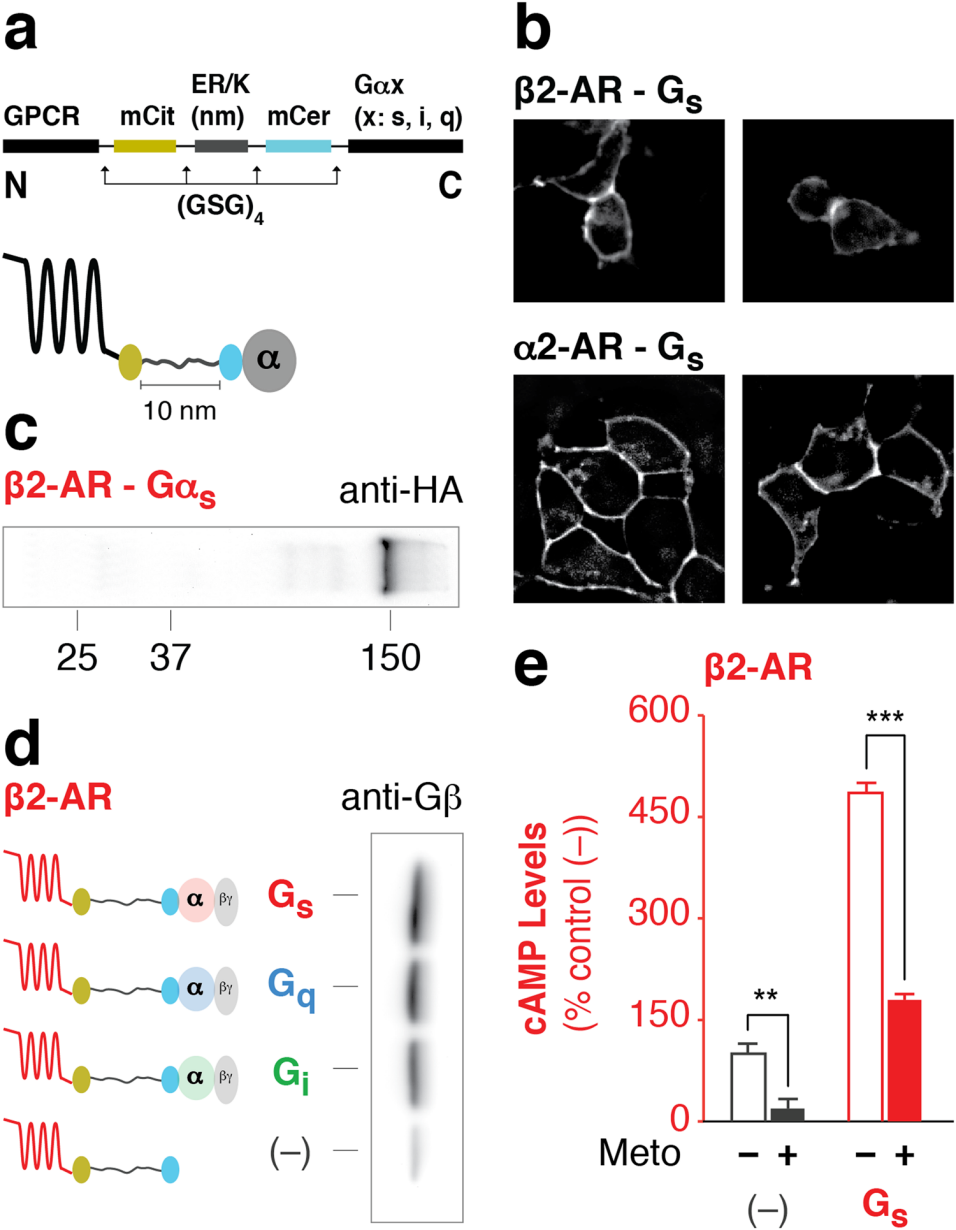
GPCR-G protein fusion sensors are intact and functional. (**a**) Schematic of the GPCR-G protein sensor design. Protein domains are separated by (GSG)_4_ linkers to ensure rotational freedom. Control (–) sensors do not contain a Gα subunit. (**b**) Sensors localize to the plasma membrane in live HEK293T cells as shown in representative images. (**c**) Western blot of membranes expressing HA-β2-AR-Gαs probed with anti-HA antibody. A distinct 150 kDa band indicates intact sensor expression. (**d**) Membranes expressing HA-β2-AR-Gαx sensors were subjected to HA-affinity purification and probed with anti-Gβ antibody. Equivalent amount of sensor is loaded per lane as assessed by mCitrine fluorescence. Gβ associates with the Gαs, Gαi, or Gαq subunit. (**e**) cAMP production in the presence or absence of inverse agonist (100 μM metoprolol) for β2-AR tethered with or without Gs. Data are derived from at least three independent experiments, with at least three replicates per condition. Data are represented as % no-G protein control (–) (mean ± S.E.M Student’s t-test was performed to evaluate significance **p ≤ *0*.*01* and ***p ≤ *0*.*001*).

Here, we combine the strengths of FRET and fusion proteins by leveraging the Systematic Protein Affinity Strength Modulation (SPASM) approach^14, 15^. GPCR SPASM sensors involve expression of a single polypeptide encoding a GPCR tethered to a G protein via an ER/K α-helix/linker that is flanked by a pair of FRET probes (mCitrine (FRET acceptor) and mCerulean (FRET donor)) (Fig. 1a). Unstructured (Gly-Ser-Gly)_4_ linkers are inserted in between each component to provide rotational flexibility (Fig. 1a). Similar to GPCR fusions, GPCR SPASM sensors enable control over GPCR-G protein stoichiometry and co-localization. ER/K linkers with end-to-end distance of 10–30 nm (measured along the α-helical backbone) are designed to provide adequate separation between the GPCR (~3 nm) and the G protein (~5 nm)^14, 16^. Increasing ER/K linker length from 10–30 nm has been shown to systematically decrease the effective concentration of the protein-protein interaction from 10 μM to 100 nM^14, 15, 17, 18^. We have previously reported on SPASM sensors involving fusions of GPCRs with C-terminal peptides of the Gα subunits^19–21^. Here, we describe SPASM GPCR sensors with the entire Gα subunit that can bridge the gap between the GPCR-G protein interaction and downstream signaling^10–13^.

In this proof-of-concept study, SPASM sensors are made for four GPCRs with varying C-tail lengths (8–87 amino acids). The β2-adrenergic receptor (β2-AR), α2-adrenergic receptor (α2-AR), α1-adrenergic receptor (α1-AR), and adenosine type 1 receptor (A_1_R) are each tethered to functionally distinct Gα subunits (Gαs-XL, Gαi_2_, and Gαq). Consistent with literature reports of co-expressed GPCR-G protein sensors^3, 5^, we find SPASM sensors with cognate GPCR-G protein pairs show enhanced interaction following agonist stimulation as indicated by a gain in FRET signal. Varying ER/K linker length from 10 nm to 30 nm systematically tunes the basal interaction between tethered GPCR-G protein pairs. However it only modulates second messenger response from select GPCR-G protein pairs over and above endogenous levels. Together, these features of the SPASM GPCR-G protein sensors facilitate both the detection of GPCR-G protein interactions in cells and establish their link to downstream signaling pathways.

## Results

### Integrity of GPCR-G protein fusions

The GPCR and Gα subunit were expressed as a single polypeptide fusion with a SPASM module between them (Fig. 1a). All individual protein elements are separated by (Gly-Ser-Gly)_4_ linkers to provide rotational flexibility (Fig. 1a). The SPASM module provides 1:1 stoichiometry of GPCR-Gα expression and consists of an ER/K α-helical linker flanked by a FRET pair (mCerulean/mCitrine)^14^. FRET probes enable monitoring of cellular expression and sensor integrity. Fusions lacking a G protein (–) served as controls to assess background signaling through endogenous G proteins. Together, these sensor properties facilitate pairwise comparison across fusions at matched cellular expression levels (see Methods). GPCR-G protein fusions were initially generated for the prototypical β2 adrenergic receptor (β2-AR). Fusions are expressed primarily ( > 80%; Supplementary Fig. 1) at the plasma membrane of HEK293T cells (Fig. 1b), are intact as assessed by immuno-blotting membrane preparations (Fig. 1c), and bind Gβγ subunits to equal extents (Fig. 1d). Fusions retain the ability to bind agonist, as witnessed by competitive displacement of [^3^H]DHA by isoproterenol binding to the receptor in the β2-AR-Gs fusions (K_i_ ~ 98 nM) (Supplementary Fig. 2). The calculated *K*_*i*_ is similar to previous reports of low affinity binding of isoproterenol in the absence of a high molar ratio of β2-AR to Gs (β2-AR – 1.4 pmol/mg; Gs ~ 100 pmol/mg)^22^. Hence, the ER/K linker does not promote ternary complex formation within the SPASM sensor. Consistent with the known basal activity of this GPCR, over-expression of β2-AR (1–2 pmol/mg total protein) elevates basal cAMP levels relative to the untransfected (UN) cells (Fig. 1e)^23^. Treatment with the inverse agonist metoprolol (Meto) significantly attenuates the enhanced cAMP levels, attesting to the role of exogenously expressed β2-AR (Fig. 1e). The functionality of Gs in the β2-AR-Gs fusion protein is witnessed by the ~5-fold increase in basal cAMP levels as compared to sensors expressing β2-AR alone (–) (Fig. 1e). Together, these measurements support the functional integrity of the β2-AR-Gs SPASM sensors in cells.

### Agonists modulate fusion interaction and downstream response

**Figure 2:**
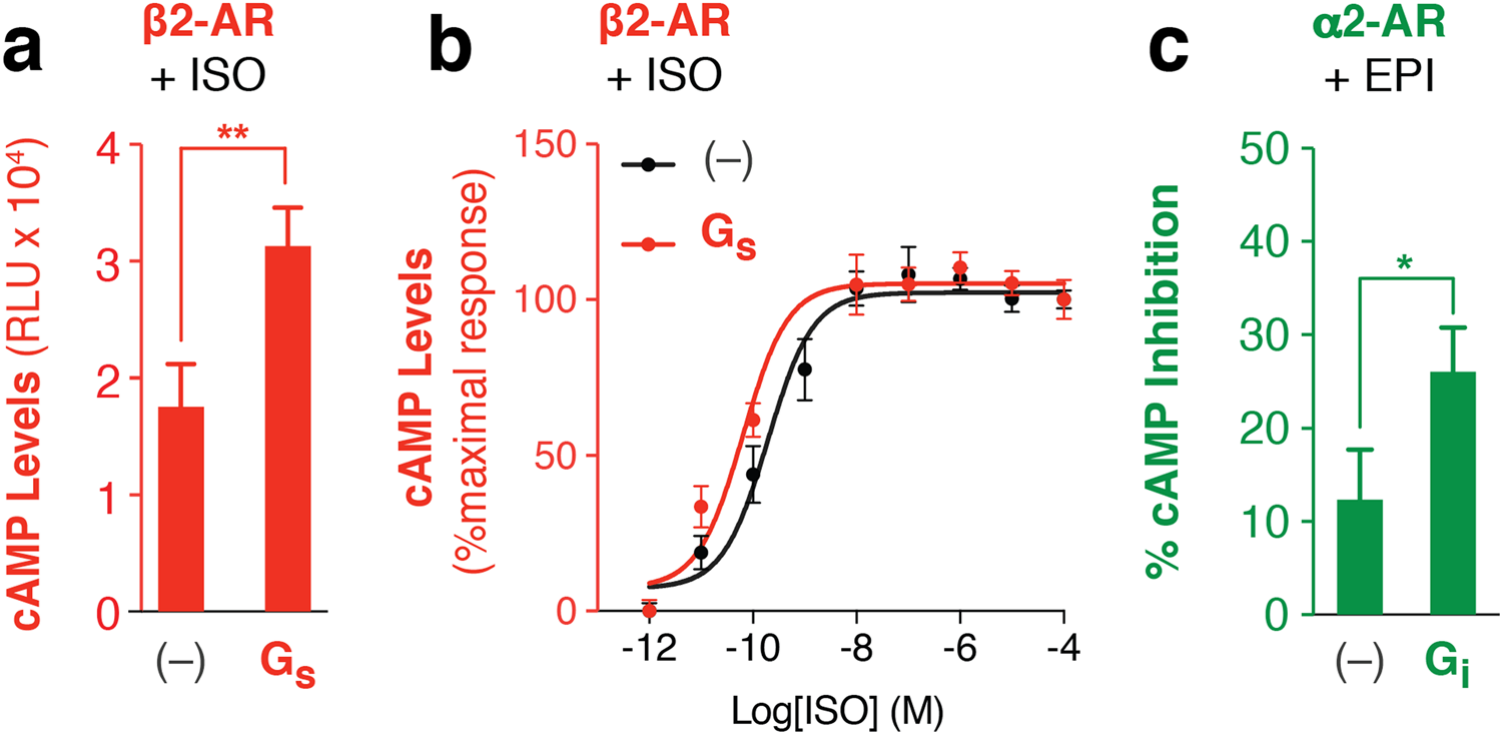
Agonist enhances downstream second messenger response via the fused G protein. (**a**) cAMP production in the presence of (**a**) saturating (100 μM) or (**b**) varying concentration of isoproterenol for β2-AR tethered with or without (–) Gs. (**b**) Data are represented as percent maximum cAMP levels for 100 μM isoproterenol treatment of β2-AR fusions tethered with or without (–) Gs. (**c**) Percent inhibition of 1 μM forskolin induced cAMP production for α2-AR fusions with or without Gi treated with 100 μM epinephrine. Data are derived from at least three independent experiments, with at least three replicates per condition (mean ± S.E.M. Student’s t-test was performed to evaluate significance *p ≤ *0*.*05*; **p ≤ *0*.*01*).

**Figure 3:**
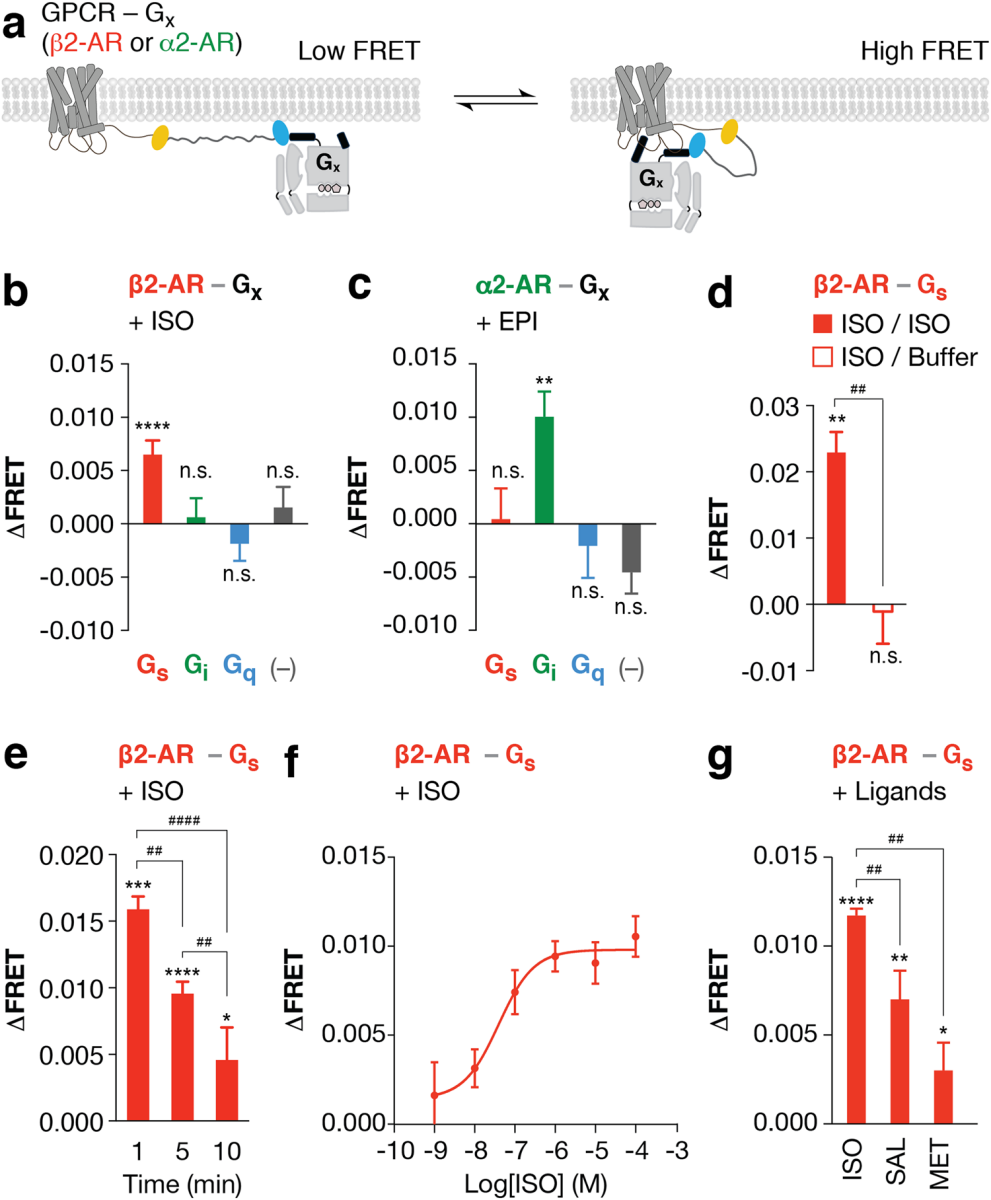
Agonist modulates cognate GPCR-Gx fusion interactions. (**a**) Schematic of the GPCR-G protein fusions linked with the 10 nm ER/K linker in the associated (high FRET) and dissociated (low FRET) states in live cells. (**b**,**c**) Change in FRET (ΔFRET) for indicated GPCR-Gx fusions following agonist treatment in live cells ((**b**) 100 μM isoproterenol (ISO) and (**c**) 1 mM epinephrine (EPI)). GPCRs are colored by their cognate G proteins; red and green indicate Gs (β2-AR) and Gi (α2-AR) coupled receptors respectively. (**d**) For ligand wash out experiments, change in FRET (ΔFRET) for cells expressing β2-AR-Gs fusion was assessed in the presence or absence of 100 μM ISO. Following addition of ISO, cells were subsequently washed with wash buffer supplemented with (filled) or without (open) 100 μM ISO. (**e**) Change in FRET (ΔFRET) for cells expressing β2-AR-Gs fusion treated with 100 μM ISO for 1, 5, or 10 minutes. (**f**) Change in FRET (ΔFRET) for cells expressing β2-AR-Gs fusion treated with varying concentration of ISO. (**g**) Change in FRET (ΔFRET) for cells expressing β2-AR-Gs fusion treated with 100 μM of β2-AR ligands (full agonist ISO, partial agonist salbutamol (SAL), and inverse agonist metoprolol (MET)). Data are derived from at least three independent experiments, with at least three replicates per condition (mean ± S.E.M. Student’s t-test was performed to evaluate significance between ligand treated and untreated conditions, with *p ≤ *0.05*; **p ≤ *0.01*; ***p ≤ *0.001*; ****p ≤ *0.0001*. One-way ANOVA with a Tukey’s post-test was performed to evaluate significance across multiple conditions, with ^##^ *p* ≤ *0*.*01*, ^*####*^ *p* ≤ *0*.*0001*).

In agreement with previous β2-AR-Gs fusions^22, 24^, tethering β2-AR to Gs with a 10 nm ER/K linker enhances both efficacy (Fig. 2a) and potency (β2-AR-Gs EC_50_ = 0.09 ± 0.05 nM; β2-AR EC_50_ = 0.44 ± 0.3 nM; Fig. 2b) of the isoproterenol induced cAMP levels compared to over-expression of β2-AR alone (–). Likewise, epinephrine treatment results in a greater loss in cAMP signaling for ER/K linked α2-AR-Gi compared to α2-AR alone (–) (Fig. 2c). To test if agonist stimulation enhances interaction between the cognate GPCR-G protein pair, changes in FRET ratio were examined. The SPASM module is designed to maintain low FRET levels in the absence of an interaction between the GPCR and G protein (dissociated state; Fig. 3a)^14^. Interaction between the GPCR and G protein (associated state; Fig. 3a) should bring the FRET donor and acceptor in closer proximity, leading to higher FRET levels. In accordance with previous studies using co-expressed GPCR and G protein FRET/BRET pair fusions, treatment with agonist (100 μM isoproterenol) results in a gain in FRET for the cognate β2-AR-Gs pairing^3, 4^, but not for non-cognate Gi, Gq, or fusions lacking a G protein (–) in live cells (Fig. 3b). Likewise, for the Gi coupled receptor, α2-AR, stimulation with 100 μM epinephrine induced a gain in FRET for Gi^4, 5^, but not Gs or Gq (Fig. 3c). ΔFRET for β2-AR-Gs = 0.006 ± 0.001 and α2-A-Gi = 0.01 ± 0.002 are small but statistically significant. These changes are comparable to previously reported measurements using co-expressed sensors of β2-AR-Gs (ΔBRET ~0.025) and α2-AR-Gi (ΔFRET ~0.022)^3–5, 25^. Agonist induced β2-AR interaction with Gs is reversible, as isoprotenerol washout returned FRET ratios to basal levels (ΔFRET −0.001 ± −0.002; Fig. 3d (open bar)). Consistent with internalization of β2-AR in HEK293 cells^26^, gain in FRET signal between β2-AR and Gs persisted up to 5 minutes and decreased following prolonged stimulation (10 minutes) with isoproterenol (Fig. 3e). Agonist stimulation led to a concentration-dependent gain in FRET between β2-AR-Gs fusion (EC50 = 39.69 nM; Fig. 3f), which paralleled the rise in cAMP production (EC_50_ = 0.09 nM; Fig. 2b). Compared with full agonist isoproterenol, at saturating concentrations (100 μM) the high affinity partial agonist salbutamol resulted in a ~63% decrease in ΔFRET for β2-AR-Gs fusion (Fig. 3g). Interestingly, metoprolol, an inverse agonist, partly suppresses β2-AR-Gs cAMP production (Fig. 1e), and displays a small but statistically significant gain in FRET (Fig. 3g). These findings suggest that metoprolol weakly facilitates an interaction between β2-AR and Gs without resulting in robust cAMP production. Together, these data suggest that the ΔFRET assay reports on the ligand-dependent change in GPCR-G protein interaction but not necessarily the activity state of the receptor or G protein.

### Fusions interact with endogenous G proteins

**Figure 4:**
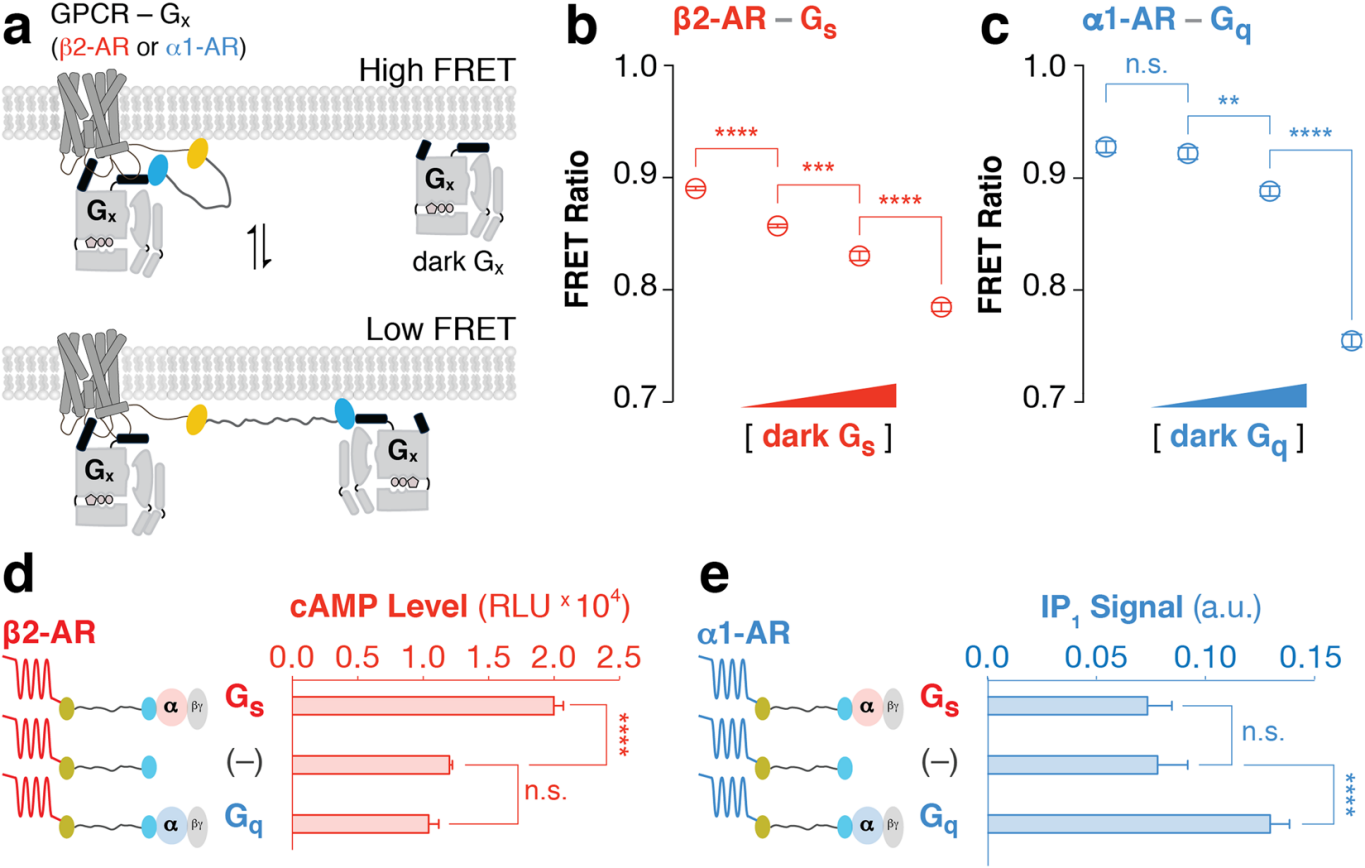
Fusions modulate GPCR-G protein signaling relative to the endogenous cellular environment. (**a**) Schematic of competitive binding of un-tethered Gx (‘dark Gx’) to GPCR-Gx fusions. (**b**,**c**) FRET Ratios (525 nm/475 nm) for cells expressing GPCR-Gx fusion and cognate Gx after transient co-transfection with different concentrations of dark Gx DNA (0, 100, 300, and 1000 ng well^−1^). GPCR-Gx fusion DNA (2–4 μg well^−1^) was optimized to maintain equivalent expression across conditions. FRET Ratios for cells co-expressing (**b**) β2-AR-Gs fusions with unlabeled dark Gs (red) or (**c**) α1-AR-Gq fusions with dark Gq (blue). (**d**) Basal cAMP and (**e**) IP_1_ levels for cells expressing (**d**) β2-AR and (**e**) α1-AR tethered to Gs, Gq, or no G protein (–). (**b**–**e**) Data are derived from at least three independent experiments, with at least three replicates per condition (mean ± S.E.M. Student’s t-test was performed to evaluate significance with *p ≤ *0.05*; **p ≤ *0.01*; ***p ≤ *0.001*; ****p ≤ *0.0001*).

One concern with fusions is that the addition of the SPASM module at the C-tail of the GPCR may cause steric hindrance between GPCR interactions with the endogenous signaling machinery. The ER/K linked fusions are designed to spatially separate the GPCR and G protein in the absence of an interaction, and should therefore freely permit interactions with non-tethered G proteins^14, 27^. To test the ability of the ER/K linked fusions to interact with non-tethered G proteins in the cellular milieu, we focused on β2-AR and α1-AR interactions with Gs and Gq, as they influence distinct second messengers (cAMP and IP_1_) and facilitate interpretation of downstream responses. First, an unlabeled (dark) Gα subunit was increasingly co-expressed relative to the ER/K linked fusions, and the FRET ratios were determined for equal levels of expression of the ER/K linked fusion (Fig. 4a). Elevated expression of Gs or Gq, relative to β2-AR-Gs or α1-AR-Gq sensors, respectively, systematically decreased FRET ratios attesting to interactions between cellular Gs or Gq and the GPCR in the ER/K linked fusions (Fig. 4b,c). Likewise, tethering the cognate G protein (Gs and Gq, respectively, for β2-AR and α1-AR) increases second messenger signaling through the cognate pathway (cAMP and IP_1_, respectively, for β2-AR and α1-AR), whereas, tethering to the non-cognate G protein (Gq and Gs, respectively, for β2-AR and α1-AR) does not suppress signaling through endogenous G proteins (Fig. 4d,e – no significant difference between sensors without G protein compared to those with non-cognate G protein). Together, these measurements demonstrate that while the ER/K linked G protein does interact with the GPCR, it does not measurably perturb interactions with endogenous components.

### ER/K linker length modulates second messenger signaling of cognate GPCR-G protein fusions

**Figure 5:**
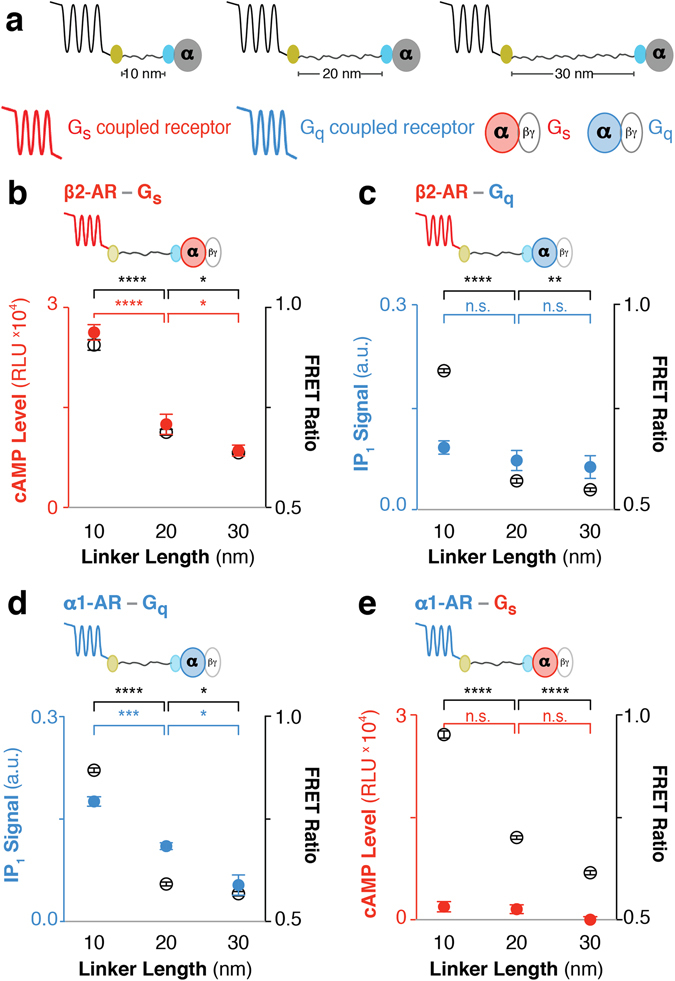
ER/K linker length specifically modulates basal β2-AR-Gs and α1-AR-Gq downstream response in live cells. (**a**) Schematic of the GPCR-G protein fusions. Color represents cognate GPCR-G protein pair and corresponding downstream response (red: β2-AR, Gs, and cAMP levels; blue: α1-AR, Gq, and IP_1_ signal). **(b**–**e**) Fusion type is indicated on the top left. FRET Ratios (525 nm/475 nm; open black circles, right y-axis) and basal (ligand-free) downstream response (filled colored circles, left y-axis) are compared to ER/K linker length (nm). (**b**) cAMP levels or (**c**) IP_1_ signal for β2-AR tethered to cognate Gs or non-cognate Gq respectively. (**d**) IP_1_ signal for cognate α1-AR-Gq fusions. (**e**) cAMP levels for α1-AR tethered to non-cognate Gs. (**b**–**e**) One-way ANOVA with a Tukey’s post-test was performed for FRET measurements (black) and downstream response (colored). Data are derived from at least three independent experiments, with at least three replicates per condition (mean ± S.E.M. *p ≤ *0*.*05*; **p ≤ *0*.*01*; ***p ≤ *0*.*001*; ****p ≤ *0*.*0001*). For additional statistical analysis see Supplementary Table 3.

**Figure 6:**
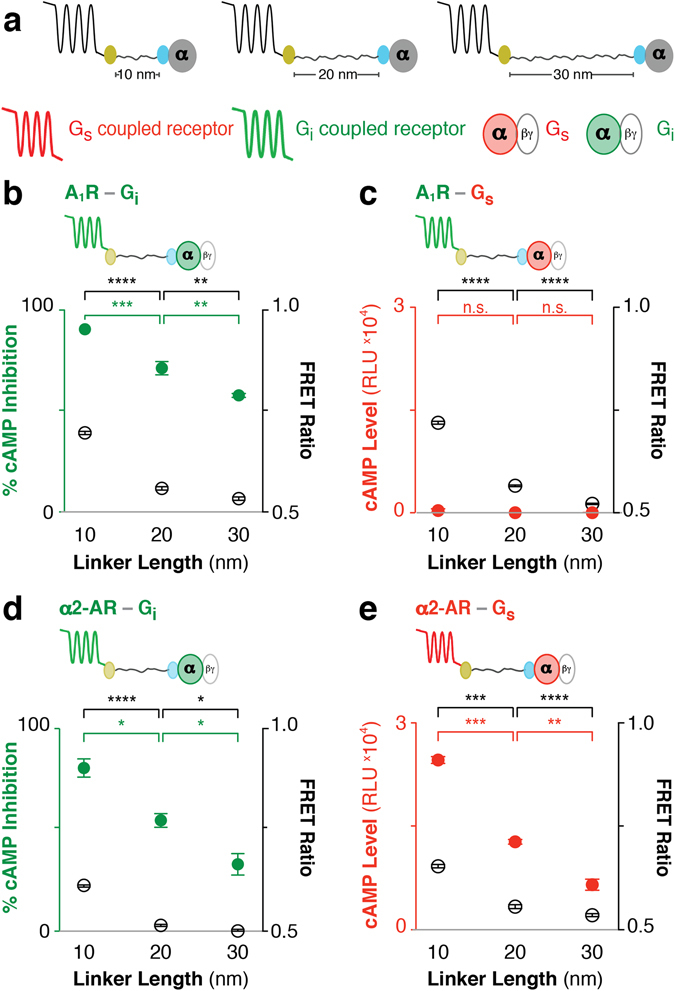
ER/K linker length tunes basal GPCR-Gi protein downstream response in live cells. (**a**) Schematic of the GPCR-G protein fusions. Color represents cognate GPCR-G protein pair and corresponding downstream response (red: α2-AR, Gs, and cAMP levels; green: A_1_R, α2-AR, Gi, and cAMP inhibition). **(b**–**e**) Fusion type is indicated on the top left. FRET Ratios (525 nm/475 nm; open black circles, right y-axis) and basal (ligand-free) downstream response (filled colored circles, left y-axis) are compared to ER/K linker length (nm). (**b**) Percent inhibition of 1 μM forskolin induced cAMP production for canonical Gi coupled adenosine type 1 receptor (A_1_R). (**c**) Basal cAMP levels for A_1_R-Gs fusions. (**d**) % cAMP inhibition for Gi fusions and (**e**) cAMP levels for Gs fusions to α2-AR, a promiscuous Gs/Gi coupled receptor^28^. (**b**,**d**) Representative experiment with at least three replicates per condition. (**c**,**e**) Data are derived from at least three independent experiments, with at least three replicates per condition. (**b**–**e**) One-way ANOVA with a Tukey’s post-test was performed for FRET measurements (black) and downstream response (colored). Data are represented as mean ± S.E.M. *p ≤ *0*.*05*; **p ≤ *0*.*01*; ***p ≤ *0*.*001*; ****p ≤ *0.0001*). For additional statistical analysis see Supplementary Table 4.

Previous studies show that increasing ER/K linker length from 10 to 30 nm decreases the effective concentration of the tethered proteins from 10 μM to 100 nM^14^. FRET ratios are sensitive to the distance between and concentration of acceptor and donor molecules^6^. As expected, FRET ratio measurements for matched sensor expression demonstrate that increasing ER/K linker length from 10 to 30 nm, systematically decreased basal FRET ratios for a range of both cognate and non-cognate GPCR-G protein interactions (Figs 5 and 6; FRET ratios are depicted by open black circles; GPCR color indicates its cognate G protein). To test whether modulating the GPCR-G protein interaction within the ER/K linked fusion influences downstream signaling, the second messenger corresponding to the tethered G protein was measured under matched protein expression levels (Figs 5 and 6; Filled circles with colors corresponding to the second messenger; red – cAMP; green – suppression of forskolin-stimulated cAMP; blue – IP_1_). In correlation with the systematic changes in the relative GPCR-Gx fusions interaction (FRET ratios), significant modulation of second messenger levels was observed only for cognate GPCR-G protein fusions (Figs 5 and 6). Specifically, tethering β2-AR (Gs coupled receptor) to Gs modulates cAMP (Fig. 5b), whereas tethering α1-AR (Gq coupled receptor) to Gq modulates IPx (Fig. 5d). In contrast, tethering β2-AR to Gq or α1-AR to Gs modulates the GPCR-Gx interaction (FRET ratio) but not IP_1_ or cAMP levels respectively (Fig. 5c,e). Tethering A_1_R (Gi coupled receptor) to Gi suppresses forskolin-stimulated cAMP levels, whereas tethering to Gs does not enhance cAMP (Fig. 6b,c). Interestingly, α2-AR, which has been shown to couple to both Gs and Gi^28^, shows both enhanced cAMP upon tethering to Gs and suppression of forskolin-stimulated cAMP upon tethering to Gi (Fig. 6d,e), unlike previously reported α2-AR fusions^11^. Together, these measurements support the ability of ER/K linked cognate GPCR-G protein fusions to successfully modulate signaling relative to endogenous levels.

## Discussion

In this study, we demonstrate the utility of ER/K-linked fusion sensors^15^ in examining the correlation between GPCR-G protein interaction and downstream signaling. Modulating the GPCR-G protein interaction using the ER/K linker systematically varies downstream signaling from cognate GPCR-G protein interactions (Figs 5b,dand 6b,d). In contrast, GPCRs tethered to non-cognate G protein do not show detectable downstream response for the non-cognate pathway (Figs 5c,e and 6c). Instead, non-cognate GPCR-G protein pairings retain signaling through the endogenous pathways (Fig. 4d,e). Likewise, increasing the concentration of exogenously expressed unlabeled Gα subunits diminishes interactions within ER/K linked fusions (Fig. 4b,c). Hence, our data suggest that rather than enforce GPCR-G protein coupling^9, 13, 22^ or sterically hinder interactions with endogenous G proteins^10, 11, 13^ the SPASM sensors can systematically modulate signaling relative to endogenous levels.

In contrast to previous GPCR-G protein fusions that use a short unstructured linker^9–11, 13, 22, 29^, in the absence of an interaction, the structured ER/K linker provides a significant spatial separation between the GPCR and the G protein fused to its ends. The ER/K linker has been previously characterized to exist primarily in an extended α-helical conformation, with end-to-end distances of ~7 to ~22 nm for 10 to 30 nm lengths along the α-helical backbone^27^. ER/K linkers are designed to modulate the effective concentration of the intra-molecular interaction^14, 15^, where the same linker length provides matched effective concentration across different GPCR-G protein pairings. However, given the localization of both GPCRs and G proteins on the plasma membrane, and the potential segregation of GPCRs into membrane micro-domains^30^, the measurements from any FRET/BRET based assay in live cells will have contributions from both intra-molecular interactions and those induced by the proximity of sensors on the plasma membrane^31^. Hence, measurements in each figure panel were performed with matched sensor expression, as determined by mCitrine fluorescence per unit cell density (see Methods). Despite potential limitations, the FRET ratio does decrease systematically with increasing ER/K linker length for all of the GPCR-G protein combinations tested suggesting that the ER/K linker alters the proximity and interaction between GPCR and G protein (Figs 5 and 6).

Unlike current FRET/BRET sensors that rely on the co-expression of labeled GPCR and G protein subunits^3–7, 32, 33^, the SPASM sensors enable the expression of a single polypeptide that provides 1:1 stoichiometry between the exogenously expressed GPCR and G protein. The specificity of the FRET response is validated using the prototypical receptor β2-AR, where agonist stimulation enhances interaction with its cognate Gs protein, with a parallel increase in downstream second messenger signaling (Figs 2b and 3f). Agonist stimulation provides changes in FRET comparable to those previously reported using co-expressed FRET/BRET pairs^3–5^ (Fig. 3b,c). The enhanced FRET is dose-dependent, reversible, and diminishes following prolonged treatment with agonist. (Fig. 3d–f). These observations establish the use of the ER/K-linked fusions to monitor ligand-induced changes in GPCR-G protein interactions in cells.

One of the advantages of ER/K-linked fusions is the ability to directly compare and contrast interaction and downstream signaling from distinct ligand-GPCR-G protein pairings. Our preliminary findings suggest two significant limitations in the interpretation of FRET-based measurements of GPCR-G protein interactions in cells. First, the ligand-induced changes in interaction do not necessarily correlate with the strength of downstream signaling. While the full agonist isoprotenerol elicits a larger FRET response than the partial agonist salbutamol, the inverse agonist metoprolol shows a small but significant increase in FRET signal (Fig. 3g). This paradoxical observation is consistent with previous reports of inactive GPCR-G protein complexes in cells^7^, and suggests that metoprolol facilitates a β2-AR-Gs interaction without G protein activation (Figs 1e and 3g). Second, ligand potency as measured using FRET is significantly lower than the potency of the downstream response. Isoprotenerol has an EC_50_ of 39.7 nM for FRET compared to 0.1 nM for cAMP (Figs 2b and 3f). Isoproterenol affinity for the β2-AR-Gs sensor was measured independently to be 98 nM (Supplementary Fig. 2). To gain insight into factors contributing to the discrepancy between the ligand potencies (FRET vs. downstream signaling), ligand efficacy (τ) was evaluated (see Methods)^34^. The fractional occupancy of the receptor for generating half maximal response is 3.9% (1/τ, Supplementary Table 5), indicating that only a small population of isoproterenol bound β2-AR-Gs is needed to generate maximal cAMP production. In contrast, the FRET response of SPASM sensors is expected to be linearly proportional to the fraction of G proteins interacting with the GPCR at equilibrium^14^. Hence, the proportion of GPCRs interacting with G protein will be limited, in part, by the ligand binding affinity. Accordingly, the EC_50_ of the FRET measurement is similar to the isoproterenol binding affinity calculated using the radio-ligand binding assay (Fig. 3f and Supplementary Fig. 2). Taken together, comparison of dose response curves for ligand binding, GPCR-G protein FRET, and cAMP response, for the same sensor construct reveal that dose-response curves of FRET are limited by ligand binding, and therefore cannot reliably report on efficacy of downstream signaling. While our studies clearly demonstrate limitations in inferring activity state of the GPCR from FRET, they do provide additional insight into the GPCR-G protein interaction especially when combined with complementary approaches such as radio-ligand binding and second messenger response.

GPCR-G protein SPASM sensors provide a novel approach to probe the link between GPCR-G protein interaction and downstream signaling responses. We have previously reported on ER/K linked fusions of GPCR and peptides derived from the Gα C-terminus^19–21^. Unlike the GPCR-G protein sensors, the GPCR-Gα C-terminus peptide sensors show a direct correlation between FRET and downstream signaling^19, 21^. The Gα C-terminus is a well-characterized component of the GPCR-G protein interface that inserts itself into the cytosolic groove of the activated GPCR^16, 35, 36^. Further, strong binding of the Gα C-terminus with the GPCR facilitates G protein activation, suggesting that GPCR interactions with the Gα C-terminus may provide a measure of ligand efficacy^37, 38^. We have previously reported that while metoprolol shows a paradoxical increase in a β2-AR-Gs interaction, it decreases the β2-AR interaction with Gαs C-terminus while enhancing the interaction with the Gαi C-terminus, with a corresponding increase in downstream Gi signaling^21^. Hence, the GPCR-G protein and GPCR-Gα C-terminus sensors provide complementary tools for dissecting the interaction and signaling state of the GPCR.

## Methods

### Reagents and buffers

Fibronectin, guanosine 5′-diphosphate sodium salt (GDP), 3-isobutyl-1-methylxanthine (IBMX), (−)-isoproterenol (+)- bitartrate salt, forskolin, and metoprolol tartarate were purchased from Sigma-Aldrich. Salbutamol hemisulfate was acquired from Tocris. *n*-dodecyl-β-D-maltopyranoside, Anagrade (DDM) was bought from Anatrace. Anti-Gβ antibody (SC378) and anti-HA antibody (MMS-101p) were purchased from Santa Cruz Biotechnology and Covance respectively. cDNAs for α1_A_-AR isoform 3 (*Homo sapiens*), and α2_A_-AR (*Sus scrofa*) were kind gifts from Dr. Richard Neubig. cDNAs for human β2-AR, long splice variant of Gαs, Gαi_2_ isoform 1, and Gαq were purchased from GE (Open Biosystems). Human A_1_R was acquired from DNASU Plasmid Repository. DNA transfection reagents X-tremeGENE HP and Mirus-LT DNA were acquired from Roche and Mirus respectively. Buffer A is 20 mM HEPES, 5 mM KCl, 145 mM NaCl, 2 mM CaCl_2_, 1 mM MgCl_2_, 0.2% dextrose (w/v), 1.5 μg mL^−1^ aprotinin and 1.5 μg mL^−1^ leupeptin at pH 7.45. Buffer B is 20 mM HEPES, 0.5 mM EDTA, 5 mM MgCl_2_, 0.1 mM DTT, 10 μM GDP, 1.5 μg mL^−1^ aprotinin, 1.5 μg mL^−1^ leupeptin and 50 μg mL^−1^ phenylmethanesulfonyl fluoride (PMSF) at pH 7.4. Buffer C is 20 mM HEPES, 0.5% decaethylene glycol monododecyl ether (C_12_E_10_), 100 mM NaCl, 1 mM MgCl_2_ 10 μM GDP, 5.5 mM β-mercaptoethanol, 10 μg mL^−1^ PMSF at pH 8.0. Buffer D is Phosphate Buffered Saline (PBS pH 7.4; Gibco^TM^), 800 μM ascorbic acid, and 0.2% dextrose (w/v).

### Molecular cloning

PCR products for α1_A_-AR (isoform 3), β2-AR, A_1_R, long splice variant of Gαs, Gαi_2_, and Gαq were obtained from human cDNA. For mammalian HEK293 expression, all GPCR and Gα constructs were cloned into a PCDNA5/FRT vector. A modular scheme was designed for cloning of GPCR sensors. Each GPCR-G protein sensor contained from N- to C-terminus: a full length GPCR, mCitrine (FRET acceptor), 10, 20 or 30 nm ER/K linker, mCerulean (FRET donor), and a Gα subunit. A (Gly-Ser-Gly)4 linker was inserted between all protein domains as part of the primer sequence to allow for free rotation between domains. Control GPCR sensors (–) did not contain a Gα subunit after mCerulean and instead had repeating (Gly-Ser-Gly)_4_ residues. All β2-AR-sensors also contained either an N-terminal HA-tag or a His-tag. All constructs were confirmed by sequencing. Peptide sequences for the 10, 20, and 30 nm ER/K linkers were described previously^14^. Briefly, the 10 nm ER/K linker is derived from *Sus scrofa’s* Myosin VI protein with the following amino acid sequence: EEEEKKKQQEEEAERLRRIQEEMEKERKRREEDEQRRRKEEEERRMKLEMEAKRKQEEEERKKREDDEKRKKK. 30 nm linker is derived from the Kelch-motif family protein (*Trichomonas vaginalis*):EEEEKKKEEEEKKQKEEQERLAKEEAERKQKEEQERLAKEEAERKQKEEEERKQKEEEERKQKEEEERKLKEEQERKAAEEKKAKEEAERKAKEEQERKAEEERKKKEEEERLERERKEREEQEKKAKEEAERIAKLEAEKKAEEERKAKEEEERKAKEEEERKKKEEQERLAKEKEEAERKAAEEKKAKEEQERKEKEEAERK. The 20 nm ER/K linker contains the first 130 residues from the 30 nm ER/K linker used in this study.

### Mammalian cell preparation and sensor expression

HEK293T-Flp-In (HEK293T, Invitrogen) cells were cultured in DMEM supplemented with 10% FBS (v/v), 4.5 g L^−1^ D-glucose, 1% Glutamax, 20 mM HEPES, pH 7.5 at 37 °C in a humidified atmosphere at 5% CO_2_. HEK293T cells were plated into 6-wells tissue culture treated plates at ~30% confluence. Cells were transfected 16–20 h later with X-tremeGENE HP DNA transfection reagent. Transfection conditions including the amount of DNA (1.4–4 μg DNA + 4.2–6 μl reagent) and the length of transfection (control sensors: 18–24 h; Gα sensors: 22–32 h) were optimized to consistently yield equivalent levels of sensor expression across different conditions. For GPCR-G protein competition assays, 100 ng, 300 ng, or 1 μg of dark Gαx were co-transfected with 2 or 4 μg of indicated sensors. For all experiments sensor integrity, localization, and sensor expression per optical density (O.D.) were tracked to ensure consistency. Experiments were conducted at 60–80% transfection efficiency evaluated by 20x and 40x magnification on a Nikon tissue-culture microscope enabled with fluorescence detection. Additionally, at the time of the experiment, 60–90% of transfected cells expressed predominately plasma membrane localized sensor with minimal localization to the intracellular compartments. Each experiment was performed at equivalent sensor expression and matched O.D. of the cell suspension using the following steps. First, cells were resuspended by gentle pipetting into their original media. Cells were then spun down (300 g, 3 min) and washed once with Buffer A. Subsequently, cells were resuspended in an appropriate volume of Buffer A to reach a 0.3 O.D. (A_600 nm_, BioMate 3 S Spectrophotometer, Thermo Scientific, 3 mm path-length, optical glass cuvette) for all fluorescence-based measurements. Finally, sensor expression was measured by mCitrine fluorescence. mCitrine fluorescence was held within 1.6–2.4 × 10^6^ counts-per-second (c.p.s) for a cell O.D. of 0.3. For each experiment, sensor integrity was tracked by measuring the mCitrine (excitation 490 bandpass 8 nm; emission range 500–600 bandpass 4 nm; emission maximum 525 nm) to mCerulean fluorescence ratio (excitation 430 bandpass 8 nm; emission range 450–600 bandpass 4 nm; emission maximum 475 nm). As part of the sensor design, mCitrine and mCerulean label the C-terminus of GPCR and N-terminus of Gα subunit respectively. All experiments were conducted at mCitrine to mCerulean fluorescence ratio of 1.7–2.1.

### Live cell microscopy and image analysis

HEK293T cells were plated (~15–20% confluence) on 35 mm glass bottom dishes (MatTek Corp) coated with 10 μg mL^−1^ fibronectin. 14–16 h after plating (30–40% confluence), cells were transfected with Mirus-LT or XtremeGENE HP DNA transfection reagent. 16–22 h post-transfection cells were washed three times with Buffer A (37 °C) to remove excess phenol red from the media. Cells were subsequently imaged for no more than 30 min in Buffer A media. Cell images were collected at 60x magnification using a Nikon TiE microscope equipped with a mercury arc lamp, 60x and 100 × 1.4 Numerical Aperture Plan-Apo oil objectives and with an Evolve 512 × 512 EM-Charge-Coupled-Device camera (Photometrics). Z-stack images were taken with 1 μm steps and the resultant stack of images was deconvolved using AutoQuantX software. Membrane expression in images was analyzed in ImageJ (NIH) using the threshold and measure tools to select and quantify membrane expression compared to internal localization. (Supplementary Fig. 1).

### Fluorescence measurements

Using FluoroMax-4 fluorometer (Horiba Scientific), FRET spectra were generated by exciting cells at 430 nm (bandpass 8 nm) in an optical glass cuvette (3–3.30-SOG-3, Starna Cells Inc.). Emission was scanned from 450–600 nm (bandpass 4 nm). For mCitrine-only measurements, cells were exited at 490 nm (band pass 8 nm), and emission was recorded from 500–600 nm (bandpass 4 nm), emission maximum was set at 525 nm.

### Live cell FRET calculations

FRET measurements were conducted at matched O.D. in Buffer A. Untransfected and transfected cells were resuspended in Buffer A at 0.3 O.D. To correct for scattering, FRET emission spectrum of an untransfected cell suspension, at equivalent O.D., was subtracted from FRET spectrum of the transfected cell suspension. The corrected FRET emission spectra were normalized to mCerulean emission (475 nm). FRET ratio was then calculated by dividing mCitrine emission (525 nm) by the mCerulean emission (475 nm).

### ΔFRET experiments

Live cell ΔFRET experiments were conducted as previously described^21^. Briefly, cells were prepared and re-suspended into pre-warm Buffer A. 90 μL aliquots of cells were added into eppendorf tubes placed in a 37 °C water bath. Samples were treated with 10 μL of indicated ligand or buffer control for 1, 5, or 10 min at 37°C. Separate and clean cuvettes were used to collect FRET spectra for treated and untreated samples to prevent cross-contamination. Change in FRET (ΔFRET) was calculated as FRET_ligand_ – FRET_buffer_.

### Reversibility of agonist-induced ΔFRET

Cells were prepared as described in the cell preparation section. First, the untreated FRET ratio was measured. Untreated cells were spun down (600 g, 1 min), washed three times, and re-suspended into 400 μL of Buffer A. FRET spectra were serially collected for 90 μL aliquots. Next, a separate well was harvested into Buffer A supplemented with 100 μM isoproterenol. Cells were subsequently spun down (600 g, 1 min) and washed three times with 400 μL of Buffer A supplemented with 100 μM isoproterenol. The spin and wash steps were completed within 5 minutes. ΔFRET was calculated by subtracting the untreated FRET ratios from the FRET ratios measured in the isoproterenol treated conditions. To assess reversibility of the isoproterenol induced ΔFRET response, cells were incubated in Buffer A supplemented with 100 μM isoproterenol for 3 minutes in a 37°C water bath. Cells were spun down (600 g, 1 min) and washed three times, and re-suspended into 400 μL of Buffer A. FRET spectra were serially collected for 90 μL aliquots. ΔFRET was calculated by subtracting the untreated FRET ratios from the FRET ratios measured in the isoproterenol wash out conditions. mCitrine:O.D. ratio was kept consistent across all conditions to ensure that each FRET measurement was taken at consistent sensor expression levels at a fixed density of cells.

### Membrane preparation

24 or 48 h post transfection (XtremeGene HP) HEK293T cells expressing indicated sensors were collected and washed once with cold PBS buffer containing 1.5 μg mL^−1^ aprotinin and 1.5 μg mL^−1^ leupeptin. Where appropriate, steps were carried out on ice and/or at 4 °C. Cells were incubated in hypotonic lysis buffer (20 mM HEPES, 0.5 mM EDTA, 0.1 mM DTT, 1.5 μg mL^−1^ aprotinin, 1.5 μg mL^−1^ leupeptin and 50 μg mL^−1^ phenylmethanesulfonyl fluoride, pH 7.4) for 5 min. Cells were lysed with a 1 mL syringe in a pre-chilled cell homogenizer (Isobiotec) using an 8-micron clearance. Lysed cells were spun at 500 g for 5 min at 4 °C. Supernatants containing cell membranes were spun at 45,000 rpm in an ultracentrifuge for 45 min at 4 °C. Membrane pellets were washed 3x with pre-chilled suspension buffer containing 20 mM HEPES, 0.5 mM EDTA, 5 mM MgCl_2_, 0.1 mM DTT, 3 μM GDP, 1.5 μg mL^−1^ aprotinin, 1.5 μg mL^−1^ leupeptin and 50 μg mL^−1^ phenylmethanesulfonyl fluoride at pH 7.4. For radioligand binding assays, membranes were washed 3x with cold resuspension buffer supplemented with 100 mM NaCl. Membranes were resuspended in 1 mL of indicated pre-chilled buffer using a rotary pestle (Fisherbrand, 30 s). Membranes were spun again at 45,000 rpm for 45 min at 4 °C. Membrane pellets were resuspended in their respective resuspension buffers using a dounce homogenizer. Total protein concentration (mg mL^−1^) was calculated using a DC Protein Assay (Bio-Rad). Membranes were flash frozen in liquid nitrogen and stored at −80 °C.

### Sensor purification from HEK293 cells

For anti-Gβ experiments, purification of His tag-β2-AR-G protein and control (−) sensors from HEK293T cells followed the previously published protocol^16^. Frozen membranes were thawed quickly to room temperature and briefly re-homogenized with a rotary pestle. 5% cholate buffer (5% sodium cholate in 50 mM HEPES, 3 mM MgCl_2_, 50 mM NaCl with 1 μg mL^−1^ aprotinin, 1 μg mL^−1^ leupeptin, 10 μg mL^−1^ phenylmethanesulfonyl fluoride, and 5.5 mM β-mercaptoethanol, pH 8.0) was added to a final concentration of 1% cholate. This mixture was incubated on ice for 45 min and separated by ultracentrifugation at ~105,000 g for 40 min at 4 °C. The supernatant was harvested and diluted drop wise with four volumes of Buffer C to one volume of supernatant, and was pipetted gently to mix. The diluted supernatant was added to nickel-NTA resin (Qiagen) and incubated 30 min at 4 °C with rotation. The resin was washed 3x with 500 μL Buffer C + 5 mM imidazole. The final wash was removed and the resin was brought to room temperature and eluted for 3–5 min with 150 μL of elution buffer (Buffer C + 200 mM imidazole). The eluted resin was spun down, the supernatant harvested, and measured for mCerulean and mCitrine fluorescence in a fluorometer (as described above). Samples were stored in SDS laemmli sample buffer at −80 °C.

### Western blot

For anti-HA western blot, 20 μg of membranes containing β2-AR-Gs sensor were boiled in Buffer B supplemented with Glycoprotein Denaturing Buffer (NEB) at 95 °C for 5 min. Boiled samples were subsequently treated with 500 units of PNGase F (NEB) for three hours at 37 °C. For anti-Gβ, equivalent amount of sensors, as measured by mCitrine fluorescence, were loaded on SDS-PAGE Gels (10% polyacrylamide). Gels were imaged for mCitrine fluorescence (excitation 488 nm, emission 520 nm, bandpass 40 nm) using a Typhoon Gel Imager (GE Healthcare). Gels were transferred to PVDF membranes for three hours at 300 mA. Anti-HA and anti-Gβ were blocked with 2% milk/TRIS-buffered saline with 1% Tween (TBST) for either one hour at room temperature or 4 °C overnight. Blots were then incubated with indicated primary antibody at a 1:1,000 dilution in 5% milk/TBST. For the anti-Gβ experiment, blots were washed with TBST, and incubated with 1:5,000 or 1:10,000 horseradish peroxidase (HRP) conjugated goat anti-rabbit IgG secondary antibody (0031460, Fisher Scientific) in 5% milk/TBST or 5% BSA/TBST for one hour at room temperature. Similarly for anti-HA experiments, blots were incubated with 1:10,000 Sheep anti-mouse HRP conjugated secondary antibody (GE Healthcare, NA931). All blots were developed with Immobilon Western Chemiluminescent HRP Substrate (Millipore). Blots were imaged using a ChemiDoc-It Imaging system (UVP). Images of gels and blots were prepared using ImageJ (NIH).

### Radio-ligand assays

Radio-ligand assays followed the previously published protocol^39^. Bmax values were estimated by incubation of 2.5, 5, and 10 μg of membrane with 5 nM [^3^H]-dihydroalprenolol ([^3^H]-DHA; PerkinElmer) for 90 min at room temperature in Tris-Buffered-Saline (TBS) pH 7.4. Samples were transferred to GF/C membranes pre-treated with 0.3% PEI solution in TBS, washed extensively with cold TBS, and dried over night at room temperature. Following addition of scintillation liquid (Microscint0, PerkinElmer,) radioactivity was measured using a 96-well scintillation counter (TopCount, PerkinElmer. Non-specific binding was estimated with 10 μM propranolol treatment and was < 1% of total binding. Dissociation constant (*K*_*d*_) of [^3^H]-DHA binding was determined by incubation of 7–10 pM (7–10 fmol/ml) of receptor with increasing concentrations of [^3^H]-DHA. *K*_*D*_ of [^3^H]-DHA binding was ~0.2 nM for β2-AR-Gs and control sensors. Competitive inhibition (*K*_*i*_) was assessed by incubation of 7 pM of receptor with increasing concentrations of isoproterenol (ISO) or buffer blank in the presence of 2 nM [^3^H]-DHA, for 90 min at room temperature. Radioactivity in samples for *K*_*D*_ and *K*_*i*_ experiments was measured as described above. Non-specific binding in all instances was found to be < 1%. Each experiment was done at least twice with different membrane preparations, with three separate samples prepared per condition, per experiment.

### cAMP assays

28–32 h post transfection (XtremeGENE HP) HEK293T cells expressing indicated sensor were harvested to assess cAMP levels using the bioluminescent cAMP Glo assay (Promega). Cells were gently suspended in their original media, were counted using a hemocytometer, and spun down (300 g, 3 min). Appropriate volume of Buffer D was added to reach 2 × 10^6^ cells mL^−1^ density for basal experiments or 4 × 10^6^ cells mL^−1^ density for ligand based assays. Cell suspensions were aliquoted into 96 wells round-bottom opaque plates. To assess basal cAMP production, cells were incubated in Buffer D supplemented with 0.25 mM IBMX for 15 min at 37 °C. To test isoproterenol-induced changes in cAMP levels, cells were incubated with varying concentration of isoproterenol for 3 min at room temperature. To assess Emax for cAMP production, cells were incubated with 100 μM of isoproterenol and 100 μM of forskolin for 3 min at room temperature or 37 °C. For cAMP suppression assays, cells were treated with 1 μM forskolin and with or without ligand for 15 min at 37 °C. Subsequently, cells were lysed and the protocol was followed according to the manufacturer’s recommendation (Promega). Luminescence was measured using a microplate luminometer reader (SpectraMax M5e, Molecular Devices). cAMP levels (relative luminescence unit, RLU) were evaluated by subtracting the untransfected background from the transfected conditions. Each experiment had four technical repeats per condition and was independently repeated at least three times (*N* > 3).

### IP_1_ assays

28–32 h post transfection (XtremeGENE HP) HEK293T cells expressing the indicated sensor were harvested to assess IP_1_ levels using the IP-One HTRF assay kit (Cisbio). Cells were gently suspended in their original media, counted using a hemocytometer, and spun down (300 g, 3 min). An appropriate volume of StimB buffer (CisBio: 10 mM Hepes, 1 mM CaCl_2_, 0.5 mM MgCl_2_, 4.2 mM KCl, 146 mM NaCl, 5.5 mM glucose, 50 mM LiCl, pH 7.4) was added to reach 3 × 10^6^ cells mL^−1^ density. Cells were incubated at 37 °C for 15 min. The manufacturer’s protocol was modified in order to achieve a high signal to noise ratio. 150 μL of suspension was incubated for one hour with 30 μL of lysis buffer (Cisbio), 54 μL StimB buffer, 6 μL IP_1_ conjugated to d2 dye, and 6 μL terbium cryptate-labeled anti-IP_1_ monoclonal antibody. IP_1_ FRET spectra were collected by exciting samples at 340 nm (bandpass 15 nm). Emission counts were recorded from 600–700 nm (bandpass 10 nm) using a long pass 475 nm filter (FSQ GG475, Newport). Raw IP_1_ signal was calculated from the 665 nm to 620 nm ratio. Basal IP_1_ signal was corrected by subtracting the untransfected IP_1_ ratio from cells expressing transfected sensor. For ligand experiments, data are presented as a change in raw IP_1_ ratio following drug treatment. Each experiment had four repeats per condition and was independently repeated at least three times (*N* > 3).

### Analysis of concentration-response curves and radio-ligand assays

Data were analyzed in GraphPad Prism 7.0c (Graphpad Software, Inc.) to obtain EC_50_ values for the ΔFRET and cAMP accumulation assays, and IC_50_, *K*_*i*_ *, K*_*D*_ values for radio-ligand binding assays. Sigmoidal curves from ΔFRET and cAMP experiments were analyzed using non-linear regression curve fitting using log (agonist or inhibitor) versus response (three parameters). The equilibrium dissociation constant for isoproterenol binding to β2-AR-Gs sensors (*K*_*i*_) and IC_50_ were calculated by fitting radio-ligand binding curve using non-linear regression analysis with competitive one site – fit *K*_*i*_ or competitive one site fit logIC_5O_ parameters respectively. Ligand efficacy (τ) was evaluated using the operational model of agonism as described previously. Briefly, dose response curves were fitted to the operational model of agonism using the following equation:

$$E=\frac{\,{E}_{max}\times {[A]}^{n}\times {{\rm{\tau }}}^{n}\,}{{[A]}^{n}\times {{\rm{\tau }}}^{n}+{({[A]}^{n}\times {K}_{A})}^{n}}$$

where *E*_*max*_ is the maximal cAMP response of the system assessed by treating the cells with 100 μM forskolin and 100 μM isoproterenol, *E* is the cAMP response to varying concentration of isoproterenol ([*A*]), and *n* is the slope of the transducer function that links ligand occupancy to response. *K*_*A*_ value was constrained to the respective *K*_*i*_ values derived from competitive radio-ligand binding assay (see above). 1/τ values were calculated to assess the fraction of isoproterenol-bound β2-AR-Gs fusion, which generates the half maximal cAMP response (see Supplementary Table 5).

### Statistical Analysis

Data are expressed as mean values ± S.E.M. Experiments were independently conducted at least three times, with 3–6 technical repeats per condition (*N* > 3). Statistical analysis was performed using GraphPad Prism 7.0c (Graphpad Software, Inc.) Statistical significance was performed for individual experiments using paired Student’s t-test. To assess how the data varied across experimental repeats, data were pooled and paired or unpaired Student’s t-tests were conducted to evaluate significance. One-way ANOVA with a Tukey’s post-test was performed to assess significance when evaluating comparisons between multiple conditions (Fig. 3d,e,g) or across fusions (Figs 5–6) with *p*-values *p ≤ *0.05*; **p ≤ *0.01*; ***p ≤ *0.001*; ****p ≤ *0.0001*.

## Electronic supplementary material

Supplementary Information

## References

[1] Dorsam RT, Gutkind JS (2007). G-protein-coupled receptors and cancer. Nat Rev Cancer.

[2] Hermans E (2003). Biochemical and pharmacological control of the multiplicity of coupling at G-protein-coupled receptors. Pharmacol Ther.

[3] Gales C (2005). Real-time monitoring of receptor and G-protein interactions in living cells. Nat Methods.

[4] Gales C (2006). Probing the activation-promoted structural rearrangements in preassembled receptor-G protein complexes. Nat Struct Mol Biol.

[5] Hein P, Frank M, Hoffmann C, Lohse MJ, Bunemann M (2005). Dynamics o receptor/G protein coupling in living cells. EMBO J.

[6] Lohse MJ, Nuber S, Hoffmann C (2012). Fluorescence/bioluminescence resonance energy transfer techniques to study G-protein-coupled receptor activation and signaling. Pharmacol Rev.

[7] Qin K, Dong C, Wu G, Lambert NA (2011). Inactive-state preassembly of G(q)-coupled receptors and G(q) heterotrimers. Nat Chem Biol.

[8] Milligan G (2000). Insights into ligand pharmacology using receptor-G-protein fusion proteins. Trends Pharmacol Sci.

[9] Seifert R, Wenzel-Seifert K, Kobilka BK (1999). GPCR-Galpha fusion proteins: molecular analysis of receptor-G-protein coupling. Trends Pharmacol Sci.

[10] Dupuis DS, Tardif S, Wurch T, Colpaert FC, Pauwels PJ (1999). Modulation of 5-HT1A receptor signalling by point-mutation of cysteine351 in the rat Galpha(o) protein. Neuropharmacology.

[11] Wise A, Milligan G (1997). Rescue of functional interactions between the alpha2A-adrenoreceptor and acylation-resistant forms of Gi1alpha by expressing the proteins from chimeric open reading frames. J Biol Chem.

[12] Wurch T, Colpaert FC, Pauwels PJ (2003). Mutation in a protein kinase C phosphorylation site of the 5-HT1A receptor preferentially attenuates Ca2+ responses to partial as opposed to higher-efficacy 5-HT1A agonists. Neuropharmacology.

[13] Wurch T, Pauwels PJ (2001). Analytical pharmacology of G protein-coupled receptors by stoichiometric expression of the receptor and G(alpha) protein subunits. J Pharmacol Toxicol Methods.

[14] Sivaramakrishnan S, Spudich JA (2011). Systematic control of protein interaction using a modular ER/K alpha-helix linker. Proc Natl Acad Sci USA.

[15] Swanson CJ, Sivaramakrishnan S (2014). Harnessing the unique structural properties of isolated alpha-helices. J Biol Chem.

[16] Rasmussen SG (2011). Crystal structure of the beta2 adrenergic receptor-Gs protein complex. Nature.

[17] Ritt M, Guan JL, Sivaramakrishnan S (2013). Visualizing and manipulating focal adhesion kinase regulation in live cells. J Biol Chem.

[18] Swanson CJ (2014). Conserved modular domains team up to latch-open active protein kinase Calpha. J Biol Chem.

[19] Semack A, Sandhu M, Malik RU, Vaidehi N, Sivaramakrishnan S (2016). Structural Elements in the Galphas and Galphaq C Termini That Mediate Selective G Protein-coupled Receptor (GPCR) Signaling. J Biol Chem.

[20] Semack, A., Malik, R. U. & Sivaramakrishnan, S. G Protein-selective GPCR Conformations Measured Using FRET Sensors in a Live Cell Suspension Fluorometer Assay. *J Vis Exp* (2016). 10.3791/54696 PMC5092009 27684955

[21] Malik RU (2013). Detection of G protein-selective G protein-coupled receptor (GPCR) conformations in live cells. J Biol Chem.

[22] Seifert R, Lee TW, Lam VT, Kobilka BK (1998). Reconstitution of beta2-adrenoceptor-GTP-binding-protein interaction in Sf9 cells–high coupling efficiency in a beta2-adrenoceptor-G(s alpha) fusion protein. Eur J Biochem.

[23] Chidiac P, Hebert TE, Valiquette M, Dennis M, Bouvier M (1994). Inverse agonist activity of beta-adrenergic antagonists. Mol Pharmacol.

[24] Seifert R, Wenzel-Seifert K, Gether U, Lam VT, Kobilka BK (1999). Examining the efficiency of receptor/G-protein coupling with a cleavable beta2-adrenoceptor-gsalpha fusion protein. Eur J Biochem.

[25] Vilardaga JP, Steinmeyer R, Harms GS, Lohse MJ (2005). Molecular basis of inverse agonism in a G protein-coupled receptor. Nat Chem Biol.

[26] Irannejad R (2013). Conformational biosensors reveal GPCR signalling from endosomes. Nature.

[27] Sivaramakrishnan S (2009). Combining single-molecule optical trapping and small-angle x-ray scattering measurements to compute the persistence length of a protein ER/K alpha-helix. Biophys J.

[28] Eason MG, Kurose H, Holt BD, Raymond JR, Liggett SB (1992). Simultaneous coupling of alpha 2-adrenergic receptors to two G-proteins with opposing effects. Subtype-selective coupling of alpha 2C10, alpha 2C4, and alpha 2C2 adrenergic receptors to Gi and Gs. J Biol Chem.

[29] Wenzel-Seifert K, Seifert R (2000). Molecular analysis of beta(2)-adrenoceptor coupling to G(s)-, G(i)-, and G(q)-proteins. Mol Pharmacol.

[30] Neubig RR (1994). Membrane organization in G-protein mechanisms. FASEB J.

[31] Lan TH (2015). BRET evidence that beta2 adrenergic receptors do not oligomerize in cells. Sci Rep.

[32] Hein P, Bunemann M (2009). Coupling mode of receptors and G proteins. Naunyn Schmiedebergs Arch Pharmacol.

[33] Hein P (2006). Gs activation is time-limiting in initiating receptor-mediated signaling. J Biol Chem.

[34] Black JW, Leff P (1983). Operational models of pharmacological agonism. Proc R Soc Lond B Biol Sci.

[35] Chung KY (2011). Conformational changes in the G protein Gs induced by the beta2 adrenergic receptor. Nature.

[36] Scheerer P (2008). Crystal structure of opsin in its G-protein-interacting conformation. Nature.

[37] Kaya AI (2014). A conserved phenylalanine as a relay between the alpha5 helix and the GDP binding region of heterotrimeric Gi protein alpha subunit. J Biol Chem.

[38] Dror RO (2015). SIGNAL TRANSDUCTION. Structural basis for nucleotide exchange in heterotrimeric G proteins. Science.

[39] Clark MJ, Neubig RR, Traynor JR (2004). Endogenous regulator of G protein signaling proteins suppress Galphao-dependent, mu-opioid agonist-mediated adenylyl cyclase supersensitization. The Journal of pharmacology and experimental therapeutics.

