# Supplementary material for: ER/K linked GPCR-G protein fusions systematically modulate second messenger response in cells
Source: Sci Rep. 2017 Aug 10;7:7749. doi: 10.1038/s41598-017-08029-3 (PMC5552854; doi:10.1038/s41598-017-08029-3)
Supplement: Supplementary file 1 — Supplementary Information [file 41598_2017_8029_MOESM1_ESM.pdf]

Supplementary Information

ER/K linked GPCR-G protein fusions systematically modulate second messenger response in cells

Rabia U. Malik, Matthew Dysthe, Michael Ritt, Roger K. Sunahara, and Sivaraj Sivaramakrishnan

Supplementary Figure 1. *GPCR-Gx fusion localize to the plasma membrane*. Representative image of live HEK293 cells expressing α2-AR-Gs fusion (see Methods for live cell microscopy). In this image 90% membrane localization was detected. Analysis was performed in ImageJ (NIH) using the threshold tool to select membrane (C; cyan outline) and internal expression (Y; yellow outline). Percent membrane expression was calculated by (C/(C+Y))*100%.

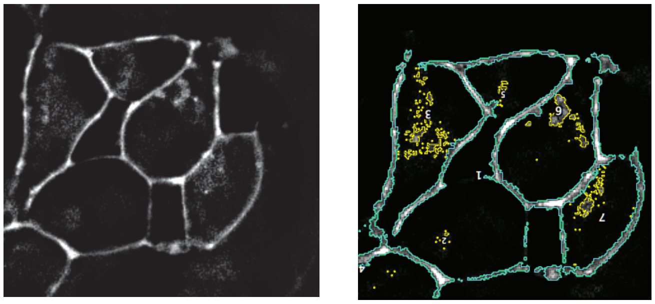

Supplementary Figure 2. *Competition by isoproterenol of [^{3}H]dihydroalprenolol binding in HEK293 membranes expressing β2-AR-Gs ER/K linked fusion*. Affinity for agonist (isoproterenol) was measured for β2-AR-Gs sensors by competitive inhibition of [^{3}H]DHA binding. Results are expressed as percent of radioligand bound in the absence of competitor.

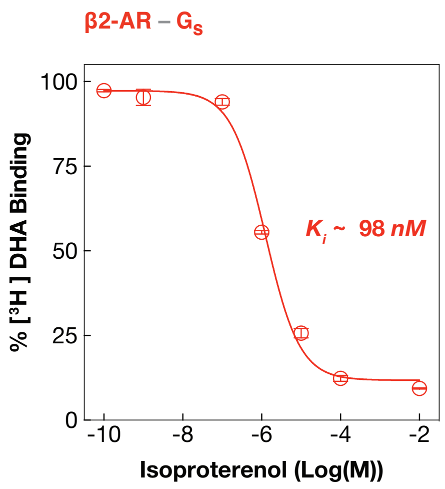

Supplementary Table 1. *Statistical analysis for β2-AR-Gx fusions*. Mean difference is the difference between the means of the indicated β2-AR-Gx (x: s or q) fusions or control sensors (–). Student's t-test (S. t-test) was performed for changes in FRET measurements for the same sensor in the presence or absence of ligand. One-way ANOVA test with a Tukey's post-test (ANOVA) was performed when comparing differences in ΔFRET across multiple sensors. A significant difference between the tested fusions are represented with the following legend: *, $p \leq 0.05$; **, $p \leq 0.01$; ***, $p \leq 0.001$; ****, $p \leq 0.0001$, n.s. not significant.

Gx protein comparison	Mean Difference	Significance	Test	Figure
s (minus) vs. s (ISO)	0.006217	****	S. t-test	Fig. 3*b* (ΔFRET)
q (minus) vs. q (ISO)	0.0003354	n.s.	S. t-test	
(–) (minus) vs. (–) (ISO)	0.001251	n.s.	S. t-test	
s (ΔFRET) vs. q (ΔFRET))	0.009624	*	ANOVA	
s (ΔFRET) vs. (–) (ΔFRET)	0.007446	*	ANOVA	

Supplementary Table 2. *Statistical analysis for α2-AR-Gx fusions*. Mean difference is the difference between the means of the indicated α2-AR-Gx (x: s or q) fusions or control sensors (–). Student's t-test (S. t-test) was performed for changes in FRET measurements for the same sensor in the presence or absence of ligand. One-way ANOVA test with a Tukey's post-test (ANOVA) was performed when comparing differences in ΔFRET across multiple sensors. A significant difference between the tested fusions are represented with the following legend: *, $p \leq 0.05$; **, $p \leq 0.01$; ***, $p \leq 0.001$; ****, $p \leq 0.0001$, n.s. not significant.

Gx protein comparison	Mean Difference	Significance	Significance	Figure
s (minus) vs s (Epi)	0.0004172	n.s.	S. t-test	Fig.3*c* (ΔFRET)
i (minus) vs i (Epi)	0.008794	**	S. t-test	
q (minus) vs q (Epi)	0.002067	n.s.	S. t-test	
(–) (minus) vs (–) (Epi)	0.004545	n.s.	S. t-test	
i (ΔFRET) vs s (ΔFRET)	0.009633	*	ANOVA	
i (ΔFRET) vs q (ΔFRET)	0.01212	*	ANOVA	
i (ΔFRET) vs (–) (ΔFRET)	0.01459	**	ANOVA	

Supplementary Table 3. *One-way ANOVA with a Tukey's post-test statistical analysis for β2-AR and α1-AR Gx fusions*. FRET Ratio and downstream response were measured for β2-AR or α1-AR fused to Gs or Gq using three different lengths ER/K linkers (10 nm, 20 nm, or 30 nm) in live cells. Mean difference is the difference between the means of the tested GPCR-Gx fusion FRET ratio or downstream response for Figure 5. A significant difference between the tested conditions are represented with the following legend: **, $p \le 0.05$; **, $p \le 0.01$; ***, $p \le 0.001$; ****, $p \le 0.0001$,* n.s. not significant.

Fusion	Assay	Comparison	Mean Difference ± S.E.M.	Significance	Figure
β2-AR-Gs	cAMP	10 vs 20 nm	13027	****	Fig. 5*b*
		10 vs 30 nm	18408	****	
		20 vs 30 nm	5380	*	
	FRET	10 vs 20 nm	0.2184	****	
		10 vs 30 nm	0.2698	****	
		20 vs 30 nm	0.05144	*	
α1-AR-Gs	cAMP	10 vs 20 nm	354.3	n.s.	Fig. 5*e*
		10 vs 30 nm	2269	n.s.	
		20 vs 30 nm	1915	n.s.	
	FRET	10 vs 20 nm	0.2514	****	
		10 vs 30 nm	0.3374	****	
		20 vs 30 nm	0.086	****	
β2-AR-Gq	IP_1	10 vs 20 nm	0.0191	n.s.	Fig. 5*c*
		10 vs 30 nm	0.03149	n.s.	
		20 vs 30 nm	0.01239	n.s.	
	FRET	10 vs 20 nm	0.2688	****	
		10 vs 30 nm	0.2904	****	
		20 vs 30 nm	0.02158	**	
α1-AR-Gq	IP_1	10 vs 20 nm	0.05501	***	Fig. 5*d*
		10 vs 30 nm	0.09868	****	
		20 vs 30 nm	0.04366	*	
	FRET	10 vs 20 nm	0.2777	****	
		10 vs 30 nm	0.3016	****	
		20 vs 30 nm	0.02389	*	

Supplementary Table 4. *One-way ANOVA with a Tukey's post-test statistical analysis for A_1R or α2-AR Gx fusion*. FRET Ratio and downstream response were measured for A_1R or α2-AR fused to Gs or Gi using three different lengths ER/K linkers (10 nm, 20 nm, or 30 nm) in live cells. Mean difference is the difference between the means of the tested GPCR-Gx fusion FRET ratio or downstream response for Figure 6. A significant difference between the tested conditions are represented with the following legend: **, $p \le 0.05$; **, $p \le 0.01$; ***, $p \le 0.001$; ****, $p \le 0.0001$,* n.s. not significant.

Fusion	Assay	Comparison	Mean Difference ± S.E.M.	Significance	Figure
A_1R-Gi	FRET	10 vs 20 nm	0.1375	****	Fig. 6*b*
		10 vs 30 nm	0.1628	****	
		20 vs 30 nm	0.02529	**	
	cAMP inhibition	10 vs 20 nm	19.25	***	
		10 vs 30 nm	32.75	****	
		20 vs 30 nm	13.5	**	
A_1R-Gs	FRET	10 vs 20 nm	0.1534	****	Fig. 6*c*
		10 vs 30 nm	0.1975	****	
		20 vs 30 nm	0.04407	****	
	cAMP	10 vs 20 nm	710.9	n.s.	
		10 vs 30 nm	482.4	n.s.	
		20 vs 30 nm	-228.4	n.s.	
α2-AR-Gi	FRET	10 vs 20 nm	0.09878	****	Fig. 6*d*
		10 vs 30 nm	0.1128	****	
		20 vs 30 nm	0.01398	*	
	cAMP inhibition	10 vs 20 nm	25.83	*	
		10 vs 30 nm	47.5	**	
		20 vs 30 nm	21.67	*	
α2-AR-Gs	FRET	10 vs 20 nm	0.09769	****	Fig. 6*e*
		10 vs 30 nm	0.1184	****	
		20 vs 30 nm	0.02075	**	
	cAMP	10 vs 20 nm	11873	***	
		10 vs 30 nm	18096	****	
		20 vs 30 nm	6223	**	

Supplementary Table 5. *Calculating isoproterenol efficacy (τ) for the β2-AR-Gs fusion*. Ligand efficacy (τ) was evaluated using the operational model of agonism as described previously. Briefly, dose response curves were fitted to the operational model of agonism using the following equation:

$$E = \frac{E_{max} \times [A]^{n} \times \tau^{n}}{[A]^{n} \times \tau^{n} + ([A]^{n} \times K_{A})^{n}}$$

where E_{max} is the maximal cAMP response of the system assessed by treating the cells with 100 μM forskolin (Fsk) and 100 μM isoproterenol (ISO), E is the cAMP response to varying concentration of isoproterenol ($[A]$), E_{sat} is the cAMP response to saturating concentration of isoproterenol (100 μM), and n is the slope of the transducer function that links ligand occupancy to response. K_A value was constrained to the respective K_i values derived from competitive radio-ligand binding assay (Supplementary Fig. 2). 1/τ values were calculated to assess the fraction of isoproterenol-bound β2-AR-Gs fusion, which generates the half maximal cAMP response.

Variable	Value	Source
E_{max}	100%	100 μM Fsk + 100 μM ISO
E_{sat}	61.1%	100 μM ISO (Fig. 2b)
K_A	97.7 nM	Radio-ligand binding assay (Supplementary Fig. 2)
n	0.232	Calculated from the operational model of agonism
τ	25.7	
1/τ	3.9%	
